# Modeling and Predicting Dengue Incidence in Highly Vulnerable Countries using Panel Data Approach

**DOI:** 10.3390/ijerph16132296

**Published:** 2019-06-28

**Authors:** Asim Anwar, Noman Khan, Muhammad Ayub, Faisal Nawaz, Asim Shah, Antoine Flahault

**Affiliations:** 1 Department of Management Sciences, COMSATS University Islamabad, Attock Campus, Punjab 43600, Pakistan; 2 Faculty of Finance and Banking, Ton Duc Thang University, Ho Chi Minh City 758307, Vietnam; 3 Swiss School of Public Health (SSPH+), Hirschengraben 82, 8001 Zürich, Switzerland; 4 Institute of Global Health, Faculty of Medicine, University of Geneva, CH-1202, 8001 Geneva, Switzerland

**Keywords:** climate change, dengue, panel fixed effect model, vulnerable countries

## Abstract

The spread of dengue has become a major public health concern in recent times due to alarming climate change. Using country level panel data over the 2000–2017 period, this paper examines the effects of climate change and socio-economic variables on the incidence of dengue-borne diseases in some of the most highly vulnerable countries. Empirical analysis shows a positive association between climate change and socio-economic conditions in the advent of dengue-borne diseases. We find that climate change, as measured by temperature, is proactively contributing to the spread of dengue-borne diseases. However, redressing the contributive factor behind climate change, via better awareness through education and improved public health facilitation, can assist in managing the occurrences and spread of dengue-borne diseases.

## 1. Introduction

Climate change is one of the worst global threats ever faced in human history [1]. Climate change, demonstrated by rising temperature, heavy rains, droughts and similar kinds of severe weather patterns, is adversely affecting the world. The Intergovernmental Panel on Climate Change (IPCC) has reported a comparative upward trend of 1.5–5.8 °C in the worldwide average surface temperature in the 21st century, which is higher than the increase in the temperature reported in the 20th century, i.e., 0.74 °C [2,3]. Global warming and a sudden shift in climatic conditions potentially create diverse problems for the world in the context of alarming human health conditions and challenges such as infectious diseases [4].

Recent years have witnessed a significant manifestation of dengue fever around the world [5]. Half of the world’s population has either been directly affected by the dengue virus or is vulnerable to different vector-borne diseases caused by dengue viruses [6]. This virus (DENV) is a byproduct of the mosquitos flavivirus that causes different diseases, such as dengue fever (DF), dengue shock syndrome (DSS) and dengue hemorrhagic fever (DHF). Ref. [7] has reported an alarming figure of 390 million people infected by DENV every year and, among these victims, 96 million people presented clinical symptoms while around 290 million showed hidden infection [8]. The swift outbreak and spread of dengue fever needs to be curtailed by redressing the conditions that support the spread, distribution and redistribution of the fatal virus. Dengue is a byproduct of different conditions, where high and low temperatures and population density supports the biotic of mosqutoes in the infected area [9]. Rising temperature helps the breeding of mosquitoes and precipitation helps the growth and distribution of dengue fever [10].

Temperature is a moderating factor affecting the ecological habits, competence of vector, and extrinsic incubation period (EIP) of mosquitoes for DENV [11]. Climate factors such as temperature and precipitation explicitly affect the biotics of dengue mosquitoes [12,13]. However, on the other hand, meteorological factors implicitly affect the distribution and spread of dengue infection [11,14]. Different studies have shown a moderating role of weather in spreading the dengue epidemics due to ambient temperature, rainfall, and consequent humidity [14,15]. The rise and fall of temperature directly affects the transmission of DENV as the EIP of DENV is prolonged at low temperatures (≤21 °C) [16]; when temperature is below 18 °C, DENV cannot spread. Ref. [17,18] report a direct relationship between rising temperature and the consequent rise in the incidence of dengue fever, where a daily increase of 1 °C increased the ratio of the dengue fever at a rate of 6.99%. The researchers found that higher temperatures (23–28 °C) result in very rapid viral growth and consequently a higher number of viruses. The hot weather conditions support a shorter viral incubation period and much earlier virus diffusion throughout the mosquitoes’ body to their salivary glands, yielding more infectious mosquitoes [19,20,21].

Although studies have probed the association between dengue-borne diseases and climate change, few studies have used the data covering a time-span similar to that used in this study. The particular time-span used is significant because the considered countries i.e., Bangladesh, India, Philippines, Thailand, Myanmar and Zimbabwe) are victims of the greatest effects of climate change and the adherent risks. This paper offers empirical evidence to enhance the understanding regarding the contributive role of climate change and other socio-economic variables in the outbreak of dengue-borne diseases in developing countries very severely affected by climate change as reported by German Watch Organization. The study will assist authorities in addressing the meteorological and socioeconomic factors contributing to the outbreak and spread of dengue-borne diseases, and taking concrete measures in the prediction and prevention of these diseases.

## 2. Material and Methods

### Description of Variables and Empirical Model

The study follows the empirical model of [22], which examines the relationship between climate change and infectious diseases. We extended the framework by incorporating the climate change and socio-economic variables which impact dengue incidence. The study focuses on the countries highly vulnerable to climate change in recent times. The empirical model is:

M = g (CV, D)(1)

where M is the number of dengue reported cases while CV represents the climate variable (mean temperature used as a proxy for CV) and D represents socio-economic variables, i.e., income and education. The socio-economic factors have been included in the model to ensure the validity of the results. A static model is estimated where the number of dengue reported cases is considered to be independent of the previous years’ reported cases. A vulnerable period for most infectious diseases is the time span associated with their spread, measured in days, weeks or even months. The researchers assumed that the spread of the disease is likely to reach its steady-state within a time span of a year; therefore, the static model is appropriate. Another reason for employing a static model is that the data available are annual. Hence, the static model is expressed as:

M_it_= β_o_ + β_o_temp*_it_* + δD*_it_* + ε*_it_* (2)

We have employed the panel data technique to analyze the impact of climate change, i.e., temperature, on the disease, i.e., dengue reported cases. The study employed a linear static model, assuming fixed effects. It is noteworthy to mention here that our data structure does not allow for analyzing random effects. According to [8], the fixed-effect model is an appropriate specification if the analysis is focused on a given number (*N*) of units so that statistical inference is conditional on the particular set of (*N*) unities, which in our case are 6 countries highly vulnerable to climate change (*N* = 6). On the other hand, random-effect models require the assumption of uncorrelated explanatory variables and a time-invariant unobservable component of the model, which is assumed to be random (for example, [23,24]). In other words, the random-effect model would require that units were selected randomly from a large number of possibilities, as is the case when the units are individuals or households.

The static model is elaborated below as:

(3) $$denguecase_{it}=\beta_{0}+\beta_{1}temp_{it}+\beta_{2}GDP_{it}+\beta_{3}edu_{it}+\varepsilon_{it}$$

The study uses a panel dataset where each variable in Equation (3) refers to country *i* at time *t,* where *D_it_* denotes socio-economic factors (GDP as a measure of income, education level), temp*_it_* is used as a proxy for climate change, and ε*_it_* is the error term. The study measures the number of reported dengue cases as a proxy for the dependent variable with reference to retrieved data from respective countries’ databases. Data for the variables temperature (yearly average of monthly mean temperature), GDP per capita (as a proxy for national income) and secondary school enrollment (as a proxy for national educational level) are retrieved from the World Bank dataset.

## 3. Results

**Figure 1:**
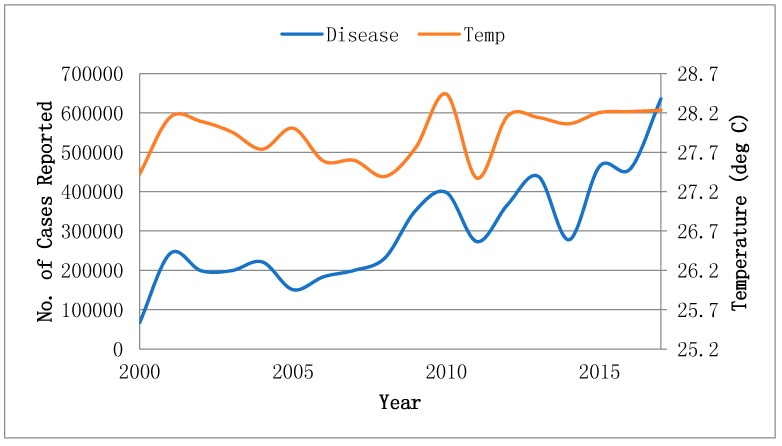
Temperature and number of overall dengue reported cases.

**Figure 2:**
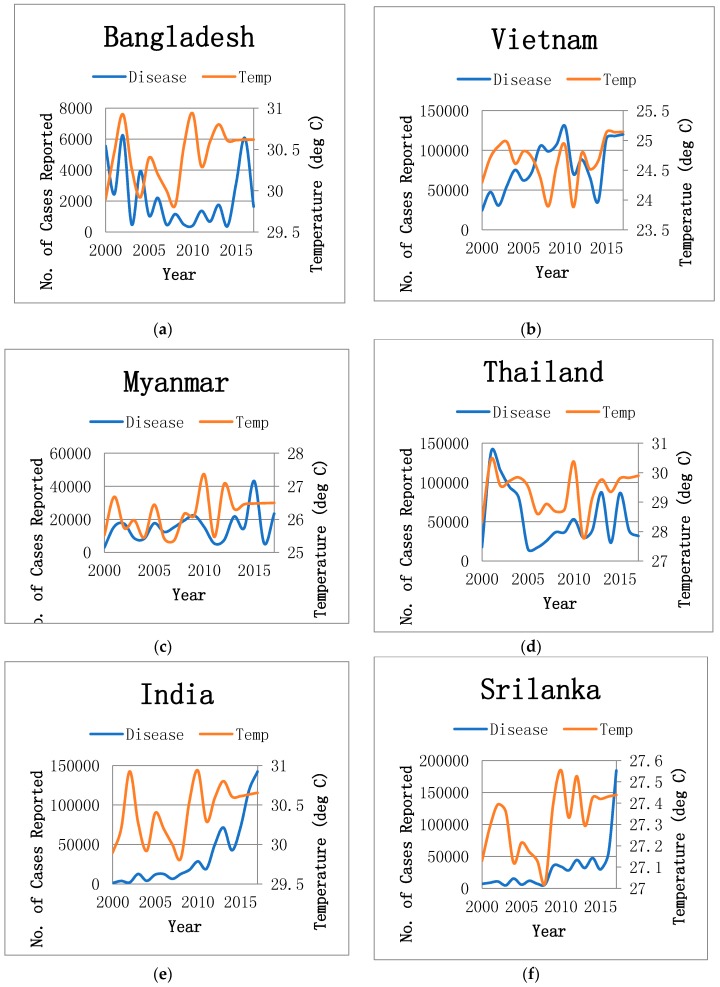
Temperature and number of dengue reported cases in individual countries. (**a**) Bangladesh, (**b**) Vietnam, (**c**) Myanmar (**d**) Thailand, (**e**) India, (**f**) Srilanka.

In 2017, 635,525 dengue infected cases from six highly affected countries were reported, which was higher in number than the 67,044 cases reported in 2000 as shown in Figure 1. The average incidence ratio in Bangladesh, Thailand, Myanmar, India, Philippine, Sri Lanka and Vietnam was −0.70, 0.80, 6.93, 114.14, 15.47, 24.57 and 3.91, respectively, over the period from 2000 to 2017, as shown in Figure 2. An upward trend in the mean temperature was found progressively in the most vulnerable countries in the recent years. The average temperature rose by 0.7 °C, 0.3 °C, 0.8 °C, 0.7 °C, 0.4 °C, 0.3 °C and 0.8 °C in Bangladesh, Thailand, Myanmar, India, Philippine, Sri Lanka and Vietnam, respectively, over the period 2000 to 2017, as shown in Figure 2. The panel fixed-effect model estimation results suggest that dengue reported cases are significantly associated with climate change measured as mean temperature, with a *p*-value of 0.01 in the six countries highly vulnerable to climate change as shown in Table 1. The results are in line with the studies of [4,19,25,26,27]. The countries’ incomes also show a positive and significant association with the incidence of dengue with a *p*-value of <0.0001, while education shows a negative and insignificant association with the incidence of dengue reported cases in the same countries, as was suggested by [28,29].

## 4. Discussion

Global warming will continue to have profound impacts on human health with reference to infectious diseases, which have undergone mushrooming growth in recent years [30,31]. This study presents the theme of dengue virus and climatic conditions, and addresses the issues of increasing ambient temperature, which causes the rapid incubation and outbreak of mosquitoes and consequent spread of the dengue virus and dengue fever. The results show that rising temperature has increased the reported cases of patients suffering from dengue fever. Climate change expedites dengue growth from a heterogeneous perspective, i.e., augmented ambient temperature expedites the dengue virus growth rate in mosquitoes by shortening the extrinsic incubation period and facilitating the transmission of mosquitoes [32]. Ambient temperature is also an active agent in regulating mosquito development, survival, and reproductive behavior [32]. The second perspective in the context of climate change is the high rate of precipitation and consequent favorable biotic conditions for mosquito incubation and the resultant outbreak of dengue borne diseases. In the present study we have found that the mean temperature might be a crucial determinant for the dynamics of dengue fever in the six most-affected countries due to climate change. DENV is a pro-climate virus that grows rapidly in rising temperatures. The EIP of mosquitoes is faster in high temperature conditions. Different studies have strongly suggested that a rise in temperature (from 23–28 °C) caused faster viral growth and magnified the level of the virus [11,33,34,35,36,37]. The hot environmental conditions also contribute to a shorter viral incubation period, and to the consequent spread of the virus throughout the mosquitoes’ body to their salivary glands much earlier, meaning more infectious mosquitoes [19].

The literature suggests a quadratic association between income and disease incidence, where the latter increases with an increase in the former, because heavy investment in the industrial sector causes climate change and the consequent outbreak of the vector borne diseases. A rise in per capita income enhances access to medical care centers and, as a result, more cases of infectious diseases are reported in well-off societies. After a certain threshold level of income, disease incidence decreases with an increase in income in the form of the health Kuznets curve. Education facilities provided to the wider population enhance awareness about dengue fever, its causes and the preventative measures that reduce the chances of its occurrence. Dengue awareness education among school and college students can assist in preventing the outbreak of dengue-borne diseases. The role of education in dengue transmission has been well-documented in the literature [28,38]. Moreover, due to inadequate financing in most of developing countries, education about dengue is not provided by formal means but through informal modes, such as verbal awareness or, to some extent, distribution of leaflets, posters or recorded material.

## 5. Conclusions

The current study focuses on presenting climate and socio-economic factors in modeling dengue incidence in the six most vulnerable countries to climate change in recent years. A dataset of dengue incidence, as measured by the number of reported cases, climate change, measured as mean temperature, education and income level for the 2000 to 2017 period was processed and used in a panel fixed-effect model. The results suggest the positive association between high temperature and dengue incidence for the panel of these most vulnerable countries. The empirical research validated the view that even minor variations in climate may change the spatiotemporal distribution of dengue fever. In addition to temperature, socioeconomic variables, such as income, have a positive impact, while the education level was shown to have a negative association with dengue incidence during the studied period. The studied countries are continuously trying to achieve industrial growth, causing climate change and the consequent advent of climate-borne diseases such as dengue. Continuous change in the global climate and the increased burden of dengue incidence in the most vulnerable countries may change the future, and evaluating the magnitude of this possible change may help in the proper future dengue resource allocation in these countries. 

Investment in the promotion of the health sector by the government and private organizations is desired to reduce the disease incidence. For that purpose, appropriate adaptation and mitigation policies need to be formulated to counter the impact of climate change on public health. The provision of health education can play a major role in providing knowledge to the people in controlling and preventing dengue fever. However, in most developing countries, the usefulness of health education is made complex by economic and political factors. 

## Figures and Tables

**Table 1:** Results of panel fixed-effect model.

Dependent Variable Disease	Coefficient	Std. Err.	*t*-Statistic	Prob
**GDP**	1.094	0.211	5.19	0.000
**Edu**	−0.253	0.367	−0.69	0.493
**Temp**	8.589	3.376	2.54	0.012
**_cons**	−29.622	10.974	−2.70	0.008
**R-square (within)**	0.277			
**N**	125			
**F-test**	0.0000			
